# Supplementary material for: The Environment Is a Key Factor in Determining the Anti-Amyloid Efficacy of EGCG
Source: Biomolecules. 2019 Dec 11;9(12):855. doi: 10.3390/biom9120855 (PMC6995563; doi:10.3390/biom9120855)
Supplement: Supplementary file 1 [file biomolecules-09-00855-s001.zip › Supplementary_Sneideris.pdf]

Supporting Information: Environment is a key factor in determining the efficacy of anti-amyloid compounds

Tomas Sneideris,[§,†] Andrius Sakalauskas,[§,†] Rebecca Sternke-Hoffmann,[§,‡] Alessia Peduzzo,[‡] Mantas Ziaunys,[†] Alexander K. Buell,[*,‡,¶] and Vytautas Smirnovas[*,†]

†*Institute of Biothechnology, Life Sciences Center, Vilnius University*

‡*Institute of Physical Biology, Heinrich-Heine-University, Düsseldorf, Germany*

¶*Department of Biotechnology and Biomedicine, Technical University of Denmark, Lyngby, Denmark*

E-mail: alebu@dtu.dk; vytautas.smirnovas@bti.vu.lt

[§]These authors contributed equally to this work

List of Figures

S1 Representative curves of insulin aggregation kinetics in the absence and presence of EGCG or $EGCG_{ox}$ under different environmental conditions.

S2 EGCG oxidation followed by UV-Vis spectroscopy.

S3 AFM images of insulin fibrils formed in PB under quiescent conditions in the absence and presence of EGCG or $EGCG_{ox}$.

S4 AFM images of insulin fibrils formed in PB under agitated conditions in the absence and presence of EGCG or $EGCG_{ox}$.

S5 AFM images of insulin fibrils formed in AC under quiescent conditions in the absence and presence of EGCG or $EGCG_{ox}$.

S6 AFM images of insulin fibrils formed in AC under agitated conditions in the absence and presence of EGCG or $EGCG_{ox}$.

S7 Evaluation of EGCG and $EGCG_{ox}$ effects on t_{50} (A) and I_{max} (B) using one-way ANOVA. *** - Significantly different (P < 0.01)

S8 Second derivative FTIR spectra of insulin amyloid-like aggregates formed in AC in the absence and presence of $EGCG_{ox}$.

S9 Effect of $EGCG_{ox}$ on t_{50} and I_{max} in AC assessed in NBS-plates and untreated-plates

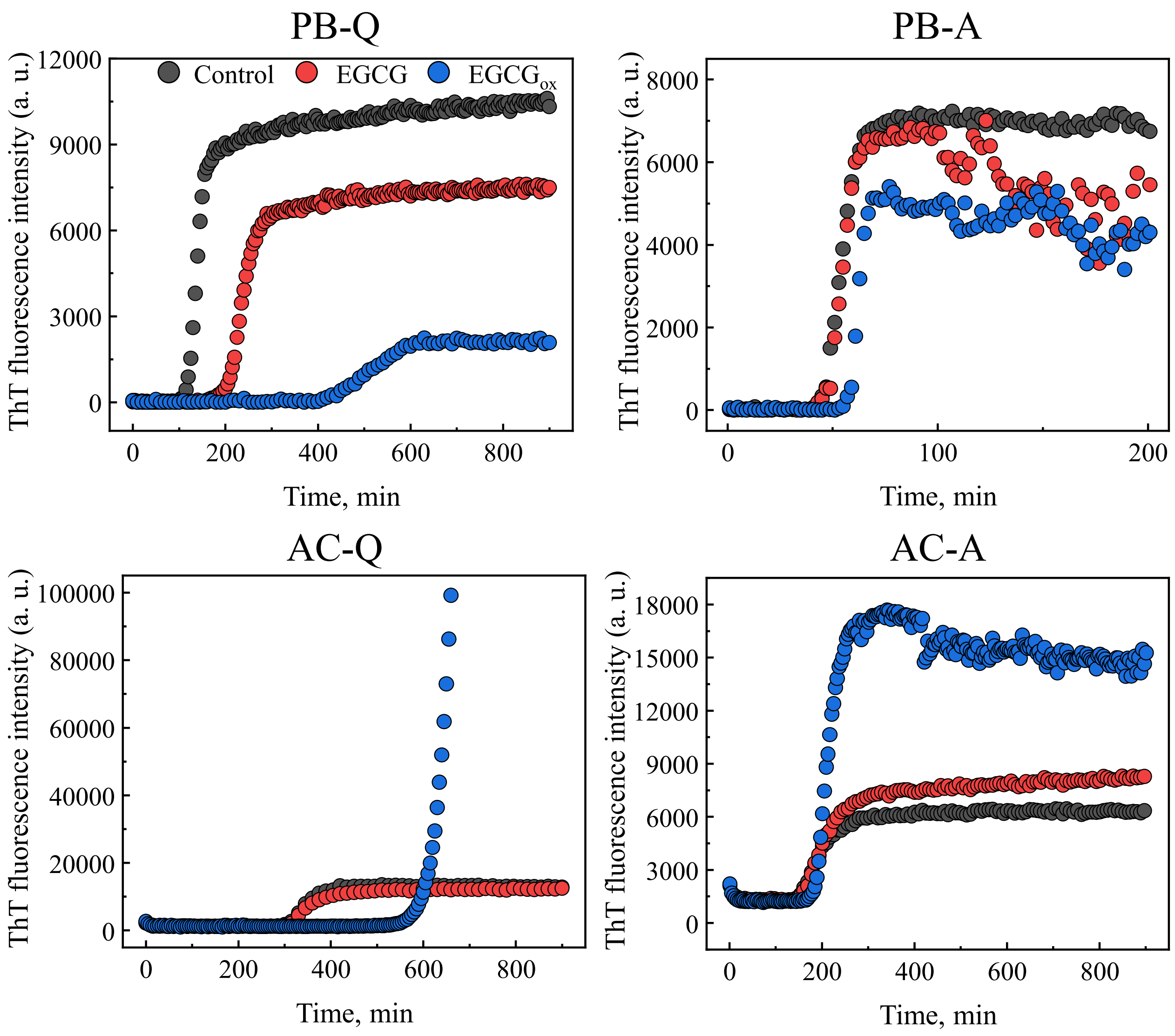

Figure S1: Representative curves of insulin aggregation kinetics in the absence and presence of EGCG or EGCG$_{ox}$ under different environmental conditions. Abbreviations PB and AC represent environmental conditions (100 mM phosphate buffer and 20 % acetic acid, respectively), while Q and A denote agitation conditions (quiescent and agitated, respectively), under which the insulin aggregation reaction was performed.

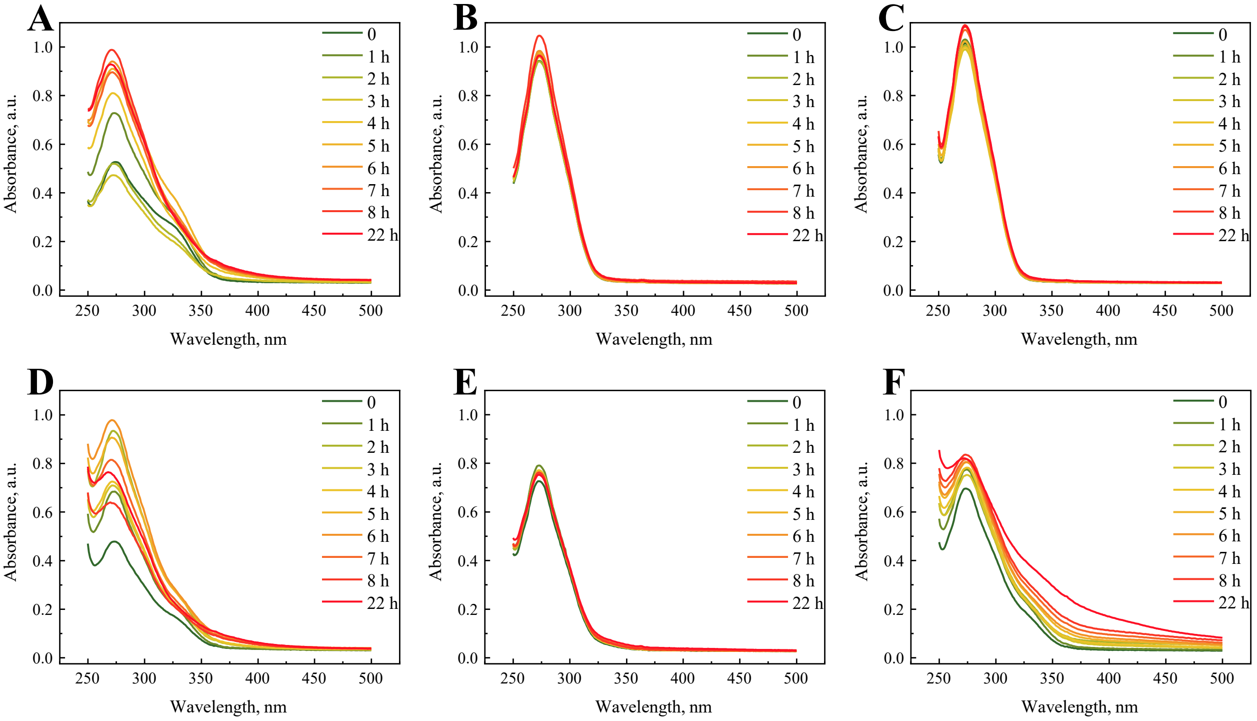

Figure S2: EGCG oxidation followed by UV-Vis spectroscopy. A) UV-Vis spectra of 10 mM EGCG in 10 mM sodium phosphate buffer pH 7.4 at 60 °C, corresponding to the conditions where a stock solution of $EGCG_{Ox}$ for the insulin experiments was produced. B) UV-Vis spectra of 172 μM EGCG in 100 mM NaCl, 100 mM sodium phosphate buffer pH 2.4 at 60 °C, corresponding to the conditions of the insulin aggregation experiments. C) UV-Vis spectra of 172 μM EGCG in 20% Acetic acid, 100 mM NaCl at 60 °C, corresponding to the conditions of the insulin aggregation experiments. D) UV-Vis spectra of 10 mM EGCG in 20 mM citric acid pH 7 at 60 °C, corresponding to the conditions where a stock solution of $EGCG_{Ox}$ for the α-synuclein experiments was produced. E), F) UV-Vis spectra of 125 μM EGCG in 150 mM citric acid pH 6 (E) and pH 7 (F) at 37 °C, corresponding to the conditions of the α-synuclein aggregation experiments.

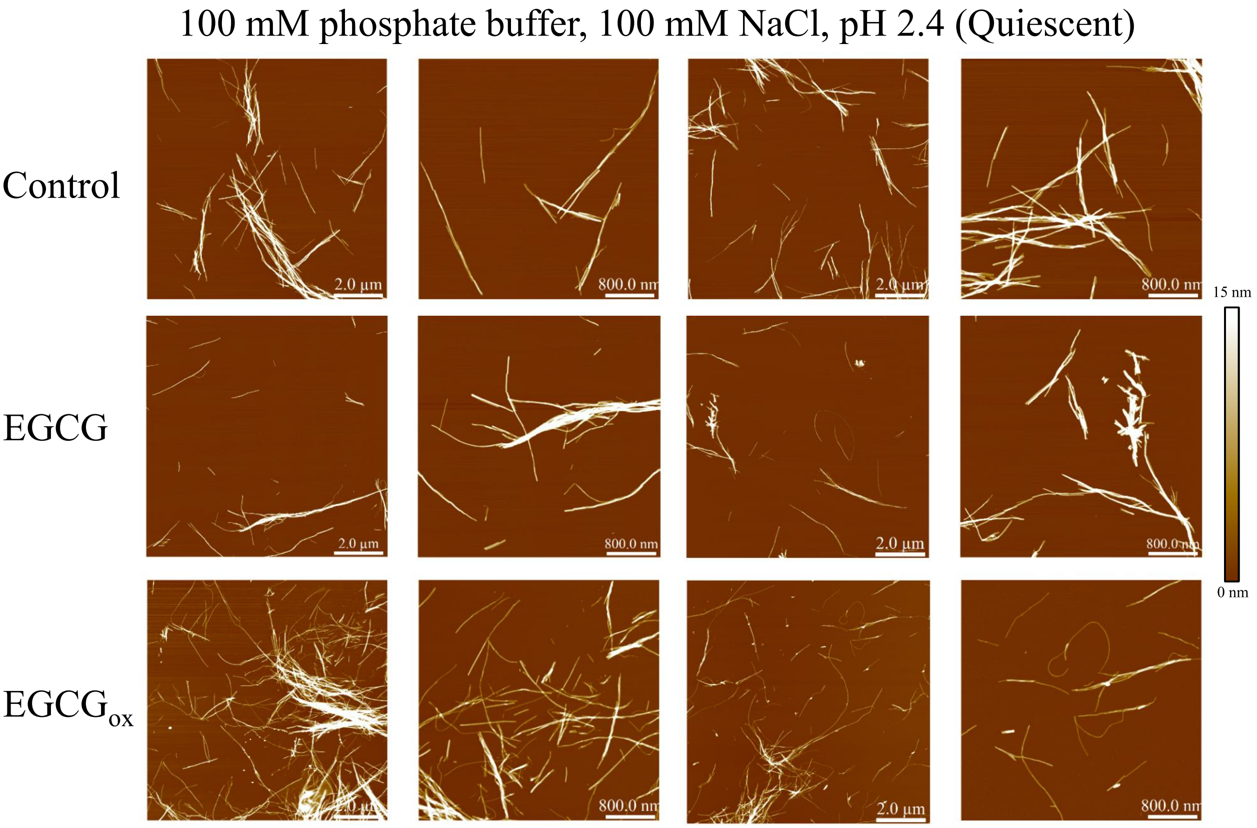

Figure S3: AFM images of insulin fibrils formed in PB under quiescent conditions in the absence and presence of EGCG or EGCG$_{ox}$.

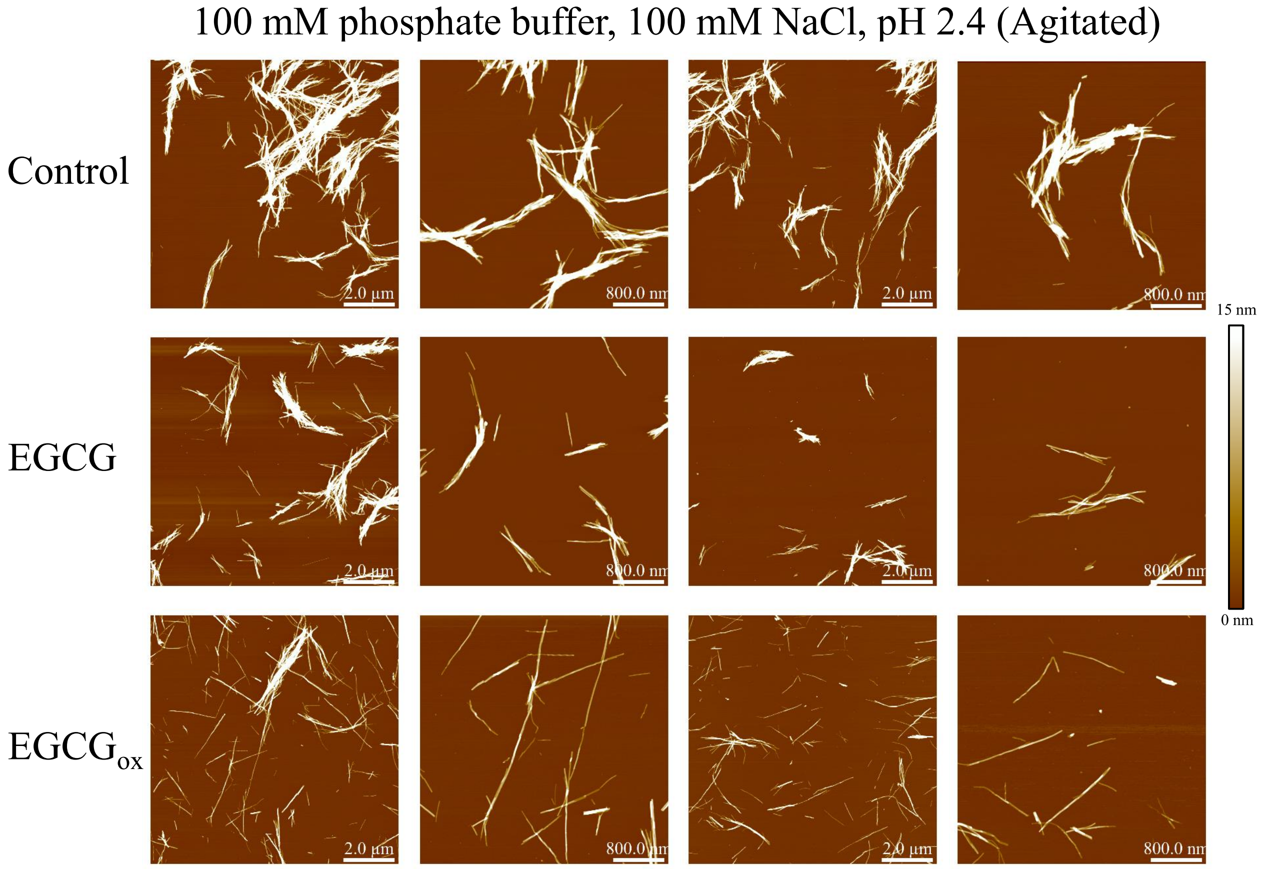

Figure S4: AFM images of insulin fibrils formed in PB under agitated conditions in the absence and presence of EGCG or EGCG$_{ox}$.

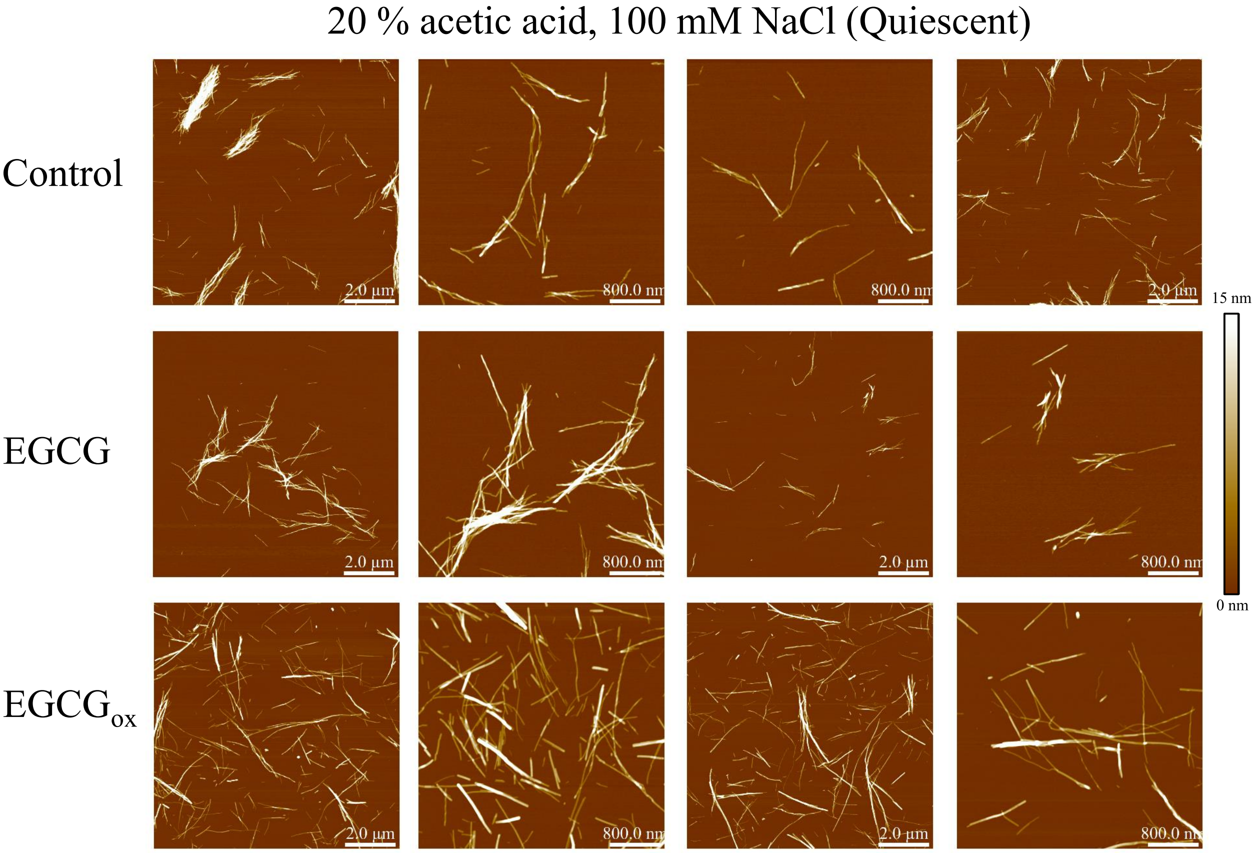

Figure S5: AFM images of insulin fibrils formed in AC under quiescent conditions in the absence and presence of EGCG or $EGCG_{ox}$.

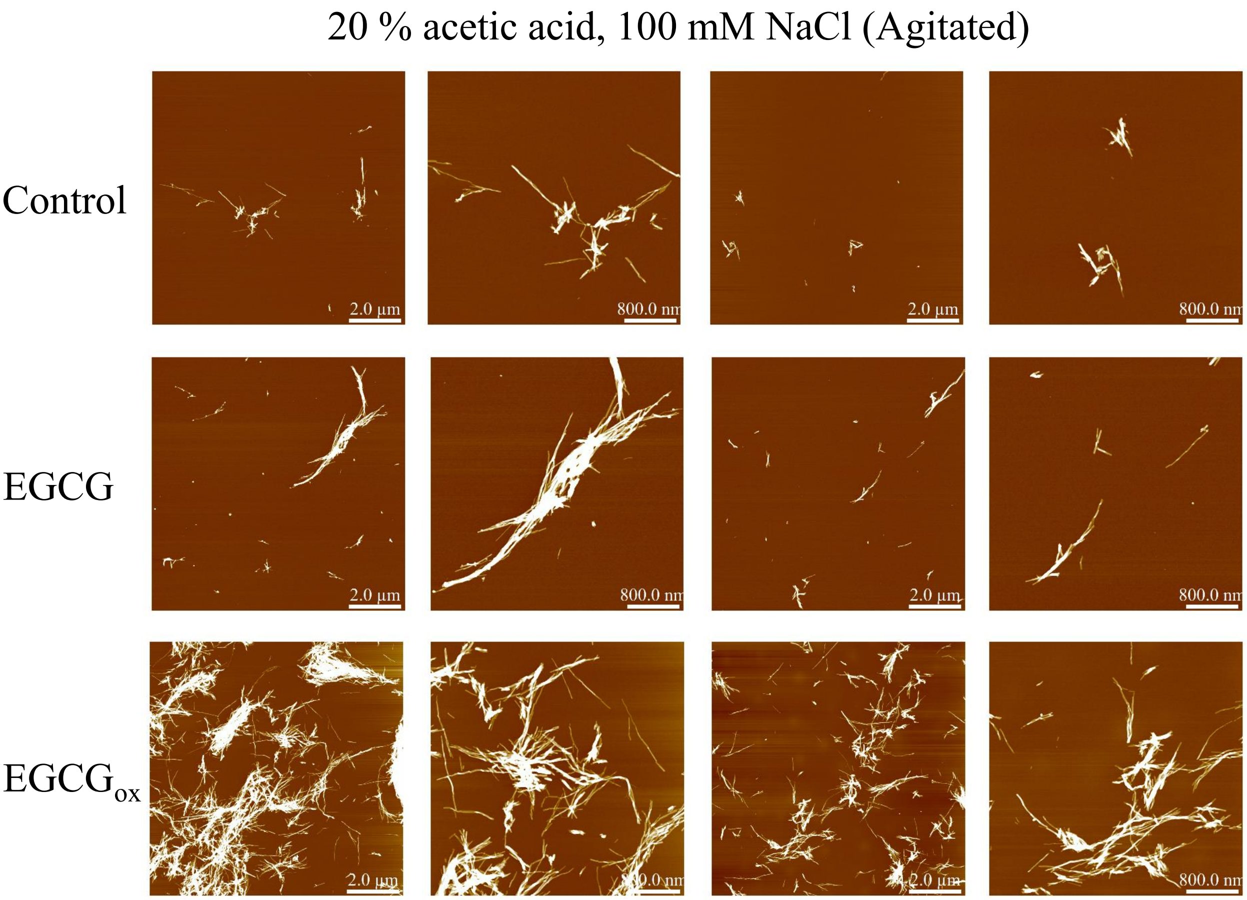

Figure S6: AFM images of insulin fibrils formed in AC under agitated conditions in the absence and presence of EGCG or EGCG$_{ox}$.

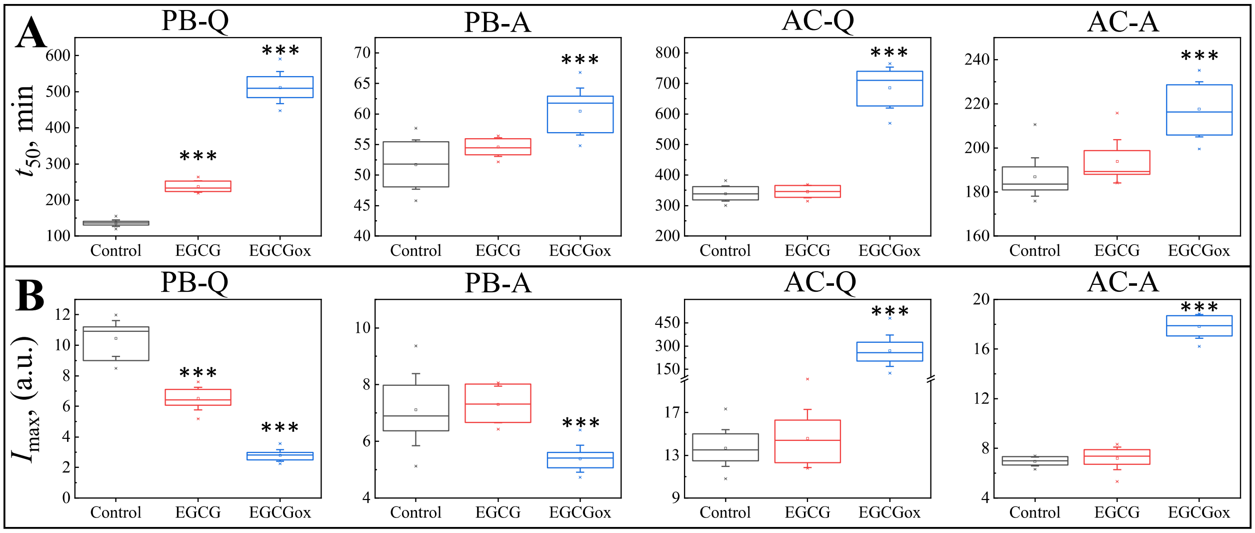

Figure S7: Evaluation of EGCG and EGCG$_{ox}$ effects on t_{50} (A) and I_{max} (B) using one-way ANOVA. *** - Significantly different (P < 0.01)

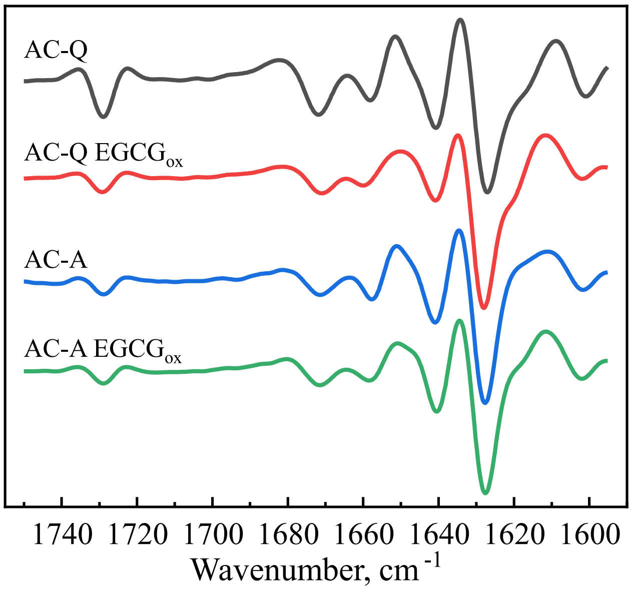

Figure S8: Second derivative FTIR spectra of insulin amyloid-like aggregates formed in AC in the absence and presence of EGCG$_{ox}$. Abbreviation AC represent environmental conditions (20 % acetic acid), while Q and A denote agitation conditions (quiescent and agitated, respectively), under which the insulin aggregation reaction was performed.

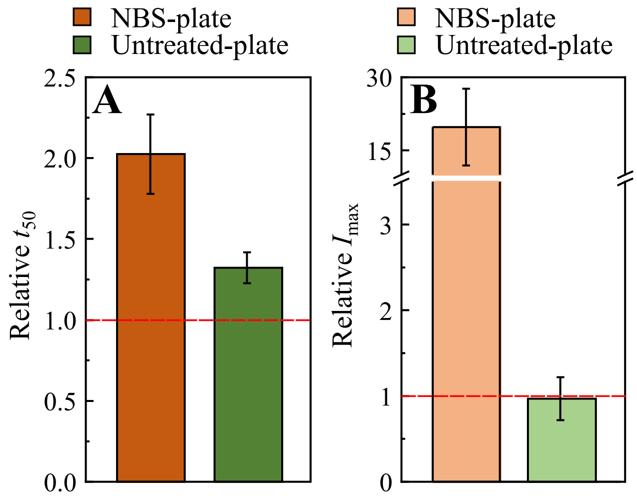

Figure S9: Effect of EGCG$_{ox}$ on t_{50} (A) and I_{max} (B) in AC assessed in NBS-plates and untreated-plates. The error bars represent standard deviations.
